# Supplementary material for: End Sequence Analysis Toolkit (ESAT) expands the extractable information from single-cell RNA-seq data
Source: Genome Res. 2016 Oct;26(10):1397–410. doi: 10.1101/gr.207902.116 (PMC5052061; doi:10.1101/gr.207902.116)
Supplement: Supplemental Material [file supp_gr.207902.116_Supp_Materials.tar.gz › ESAT-esat_v0.1/doc/html/classbroad_1_1pda_1_1countreads_1_1_fastq_library_stats.html]

ESAT: broad.pda.countreads.FastqLibraryStats Class Reference


ESAT


- Main Page
- Related Pages
- Packages
- Classes
- Files

- Class List
- Class Index
- Class Hierarchy
- Class Members

All Classes Namespaces Files Functions Variables Enumerator Pages

- broad
- pda
- countreads
- FastqLibraryStats

Public Member Functions |
Static Public Member Functions |
List of all members

broad.pda.countreads.FastqLibraryStats Class Reference

|  |  |
| --- | --- |
| Public Member Functions | |
|  | FastqLibraryStats (String singleReadFastq) throws IOException |
|  | |
|  | FastqLibraryStats (String read1fastq, String read2fastq) throws IOException |
|  | |
| int | getNumUniqueReads () |
|  | |
| double | getPercentDuplicated () |
|  | |
| int | getTotalReads () |
|  | |
| long | getEstimatedLibrarySize () |
|  | |
| void | writeSeparateFiles (String outUniquePrefix, String outDupPrefix, boolean pairedReads) throws IOException |
|  | |

|  |  |
| --- | --- |
| Static Public Member Functions | |
| static void | main (String[] args) throws IOException |
|  | |

## Detailed Description

Author
:   prussell

## Constructor & Destructor Documentation

|  |  |  |  |  |  |
| --- | --- | --- | --- | --- | --- |
| broad.pda.countreads.FastqLibraryStats.FastqLibraryStats | ( | String | *singleReadFastq* | ) | throws IOException |

Constructor for single end reads

Parameters
:   |  |  |
    | --- | --- |
    | singleReadFastq |  |

Exceptions
:   |  |  |
    | --- | --- |
    | IOException |  |

Here is the caller graph for this function:

|  |  |  |  |
| --- | --- | --- | --- |
| broad.pda.countreads.FastqLibraryStats.FastqLibraryStats | ( | String | *read1fastq*, |
|  |  | String | *read2fastq* |
|  | ) |  | throws IOException |

Constructor for paired end reads

Parameters
:   |  |  |
    | --- | --- |
    | read1fastq |  |
    | read2fastq |  |

Exceptions
:   |  |  |
    | --- | --- |
    | IOException |  |

## Member Function Documentation

|  |  |  |  |  |
| --- | --- | --- | --- | --- |
| long broad.pda.countreads.FastqLibraryStats.getEstimatedLibrarySize | ( |  | ) |  |

Get the estimated library size based on number of reads and percent duplicates

Returns
:   The estimated library size

|  |  |  |  |  |
| --- | --- | --- | --- | --- |
| int broad.pda.countreads.FastqLibraryStats.getNumUniqueReads | ( |  | ) |  |

Get the number of unique reads

Returns
:   The number of unique reads

|  |  |  |  |  |
| --- | --- | --- | --- | --- |
| double broad.pda.countreads.FastqLibraryStats.getPercentDuplicated | ( |  | ) |  |

Get the proportion of reads that are duplicates

Returns
:   The proportion of reads that are duplicated

|  |  |  |  |  |
| --- | --- | --- | --- | --- |
| int broad.pda.countreads.FastqLibraryStats.getTotalReads | ( |  | ) |  |

Get the total number of reads

Returns
:   The total number of reads

|  |  |  |  |  |  |
| --- | --- | --- | --- | --- | --- |
| static void broad.pda.countreads.FastqLibraryStats.main | ( | String[] | *args* | ) | throws IOException |

static

Parameters
:   |  |  |
    | --- | --- |
    | args |  |

Exceptions
:   |  |  |
    | --- | --- |
    | IOException |  |

Here is the call graph for this function:

|  |  |  |  |
| --- | --- | --- | --- |
| void broad.pda.countreads.FastqLibraryStats.writeSeparateFiles | ( | String | *outUniquePrefix*, |
|  |  | String | *outDupPrefix*, |
|  |  | boolean | *pairedReads* |
|  | ) |  | throws IOException |

Write fastq files of unique reads and duplicated reads

Parameters
:   |  |  |
    | --- | --- |
    | outUniquePrefix | Output fastq file of unique reads |
    | outDupPrefix | Output fastq file of duplicated reads |
    | pairedReads | Whether the reads are paired |

Exceptions
:   |  |  |
    | --- | --- |
    | IOException |  |

---

The documentation for this class was generated from the following file:

- java/broad/pda/countreads/FastqLibraryStats.java


---

Generated on Fri Aug 15 2014 15:10:58 for ESAT by  

 1.8.7
